# Dual RNA-seq to catalogue host and parasite gene expression changes associated with virulence of *T. annulata*-transformed bovine leukocytes: towards identification of attenuation biomarkers

**DOI:** 10.1038/s41598-023-45458-9

**Published:** 2023-10-24

**Authors:** Khawla Elati, Shahin Tajeri, Isaiah Obara, Moez Mhadhbi, Erich Zweygarth, Mohamed Aziz Darghouth, Ard Menzo Nijhof

**Affiliations:** 1 Institute of Parasitology and Tropical Veterinary Medicine, Freie Universität Berlin, Robert-Von-Ostertag-Str. 7, 14163 Berlin, Germany; 2 Veterinary Centre for Resistance Research, Freie Universität Berlin, Robert-Von-Ostertag-Str. 8, 14163 Berlin, Germany; 3 Laboratoire de Parasitologie, École Nationale de Médecine Vétérinaire de Sidi Thabet, Institution de la Recherche et de l’Enseignement Supérieur Agricoles, Univ. Manouba, 2020 Sidi Thabet, Tunisia; 4 Department of Veterinary Tropical Diseases, Faculty of Veterinary Science, University of Pretoria, Pretoria, South Africa

**Keywords:** Gene expression, Gene regulation, Genetic association study

## Abstract

The apicomplexan parasite *Theileria annulata* is transmitted by *Hyalomma* ticks and causes an acute lymphoproliferative disease that is invariably lethal in exotic cattle breeds. The unique ability of the schizont stage of *T. annulata* to transform infected leukocytes to a cancer-like phenotype and the simplicity of culturing and passaging *T. annulata*-transformed cells in vitro have been explored for live vaccine development by attenuating the transformed cells using lengthy serial propagation in vitro. The empirical in vivo evaluation of attenuation required for each batch of long-term cultured cells is a major constraint since it is resource intensive and raises ethical issues regarding animal welfare. As yet, the molecular mechanisms underlying attenuation are not well understood. Characteristic changes in gene expression brought about by attenuation are likely to aid in the identification of novel biomarkers for attenuation. We set out to undertake a comparative transcriptome analysis of attenuated (passage 296) and virulent (passage 26) bovine leukocytes infected with a Tunisian strain of *T. annulata* termed Beja. RNA-seq was used to analyse gene expression profiles and the relative expression levels of selected genes were verified by real-time quantitative PCR (RT-qPCR) analysis. Among the 3538 T*. annulata* genes analysed, 214 were significantly differentially expressed, of which 149 genes were up-regulated and 65 down-regulated. Functional annotation of differentially expressed *T. annulata* genes revealed four broad categories of metabolic pathways: carbon metabolism, oxidative phosphorylation, protein processing in the endoplasmic reticulum and biosynthesis of secondary metabolites. It is interesting to note that of the top 40 genes that showed altered expression, 13 were predicted to contain a signal peptide and/or at least one transmembrane domain, suggesting possible involvement in host-parasite interaction. Of the 16,514 bovine transcripts, 284 and 277 showed up-regulated and down-regulated expression, respectively. These were assigned to functional categories relevant to cell surface, tissue morphogenesis and regulation of cell adhesion, regulation of leucocyte, lymphocyte and cell activation. The genetic alterations acquired during attenuation that we have catalogued herein, as well as the accompanying in silico functional characterization, do not only improve understanding of the attenuation process, but can also be exploited by studies aimed at identifying attenuation biomarkers across different cell lines focusing on some host and parasite genes that have been highlighted in this study, such as bovine genes (CD69, ZNF618, LPAR3, and APOL3) and parasite genes such as TA03875.

## Introduction

Although many tick-borne *Theileria* species infect domestic and wild ruminants, pathogenicity in cattle is mainly confined to *Theileria annulata* and* Theileria parva*. The uniqueness of these *Theileria* species lies in the ability of their schizont stages to multiply and transform infected bovine leukocytes to a cancer-like phenotype^1^. For *T. annulata*, the acute lymphoproliferative diseases that results from infection is termed Tropical Theileriosis (TT). The disease is found throughout north Africa, the Mediterranean basin, the Middle East, India and southern Asia, where an estimated 250 million cattle (*Bos taurus*), as well as buffalos (*Bubalus bubalus*) are at risk^2^. TT is among the most serious constraints to cattle production in the countries where it is found^3–6^.

Sporozoites of *T. annulata* mature in the salivary glands of infected *Hyalomma* ticks during feeding and are transmitted to cattle along with tick saliva. In cattle, the sporozoites invade either macrophages or B cells and develop into multinucleated *T. annulata* forms named schizonts that induce hyperproliferation and dissemination of infected cells, causing a leukaemia-like disease^7^. As a result of the rapid expansion of infected bovine cell populations, the local lymph node draining the site of infection often contains large numbers of infected cells that are subsequently disseminated throughout the lymphoid system and to non-lymphoid tissues^8^. The lymphoproliferation typically results in death within three weeks of infection^9^. The mode of oncogenesis by *T. annulata* is little understood, but there is evidence that it involves substantial changes in the infected host cell transcriptome due to parasite-dependent activation of key host cell transcription factors such as Activator Protein 1 (AP-1)^10^, nuclear factor kappa B (NF-kB)^11^, E2F transcription factor^12^ and Hypoxia-inducible factor 1-alpha (HIF1-a)^13^. The transcriptional change in the host supports immortalization, constant proliferation and widespread dissemination. Several host kinases that act upstream of these transcription factors are found to be constitutively activated in presence of live intracellular schizonts, examples include c-Jun N-terminal kinase (JNK)^14^, Src kinase^15^ and phosphatidylinositol-3 kinase (PI3K)^16^. It is believed that parasite secreted factors contribute to alterations in host cell signalling pathways regulating the above mentioned kinases and transcription factors^17^. However, due to the lack of genetic tools to manipulate the *Theileria* genome, their exact roles in the transformation process remain unclear.

Because of the often acute and fatal nature of *T. annulata* infections, control of TT is challenging. The routine form of control has been the use of acaricides to kill the tick vectors. However, acaricide use is unsustainable, with limitations evident in their high costs and the continuing selection of acaricide-resistant tick populations^18,19^. Similarly, the need to treat animals during the early stages of disease and the high costs of the therapeutic compound (buparvaquone) imposes constraints on the effectiveness of chemotherapy.

Vaccination can only offer a sustainable approach to TT control in cattle if it can be shown to be efficacious and cost-effective. The ability of *T. annulata* to multiply and transform infected leukocytes has been explored to develop an immunization procedure against the disease, which involves attenuating parasitised cells by lengthy serial propagation in vitro^20^. The perception that transboundary use of attenuated *T. annulata* infected cell lines could introduce ‘foreign’ parasite genotypes and result in enhanced disease problems, led to the need to generate culture-attenuated live vaccines independently for each country using local parasite isolates^3,6,21–23^. In Tunisia, the attenuated schizont infected cell line ‘Beja’ was developed and used at passage 280 to immunize cattle under field conditions^24^. This vaccine proved to be particularly effective when applied to control TT in regions of endemic infections and thus has great potential for widespread deployment in small dairy herds in Tunisia, where this is the most common production system. However, its use in the field is hampered due to logistic issues of the delivery of the vaccine as this requires a liquid nitrogen chain and biosafety constraints due to the potential vaccine contamination with other pathogens.

A crucial part of the parasitised cell line vaccination strategy is the lengthy in vitro propagation coupled with occasional inoculation into calves to assess attenuation. A cell line is considered to be attenuated if the calves do not develop clinical signs and schizonts and piroplasms cannot be detected in lymph nodes, liver biopsies or Giemsa-stained blood smears, respectively. An attenuated line is considered as a vaccine when it has undergone challenge experiments and immunogenicity tests as well as tolerance tests with the most susceptible cattle categories in the target population (i.e. exotic dairy breeds cows in lactation/pregnancy) this in addition to microbiological and quality control tests^24^. The nature of the mechanism underlying attenuation has remained largely unclarified and could potentially involve, separately or in combination, selection of non-virulent subpopulations, genomic alterations, or changes in gene expression^25^. For example, Darghouth et al. (1996)^24^ observed that virulent low passage *T. annulata*-transformed leukocytes often contain multiple parasite genotypes compared to attenuated lines. Interestingly, it has been shown that upon vaccination, *T. annulata* schizonts are transferred from vaccine cells to leukocytes of the recipient animal conferring an attenuated phenotype on them^26^. Thus, virulence of *T. annulata* transformed leukocytes is a parasite encoded trait but it is the infected host cell phenotype that decides on the outcome of an infection; in case of vaccination with attenuated leukocytes leading to establishment of a mild subclinical infection in vaccinated animals that protects against future field challenge. Substantial molecular changes in the long-term passaged *T. annulata* transformed macrophages have been identified and characterised. A relatively well-studied example is transcriptional downregulation of bovine MMP9 that has been observed in several *T. annulata* attenuated cell lines of different geographic origins^27,28^. MMP9 is an infection induced bovine gene that is under control of AP-1^27^ and drives tissue dissemination of *T. annulata*-transformed leukocytes^29^. In attenuated macrophages dampened AP-1 activity results in reduced MMP9 expression and activity, partially explaining the diminished dissemination of attenuated cells^27^. Downregulated expression of another host factor, Transforming growth factor-beta 2 (TGF-ß2), has also been linked to reduced dissemination potential of attenuated cells^30^. Recently, downregulated expression of infection-induced microRNAs *mir-126-5p* and *mir34c-3p* was shown to contribute to reduced Matrigel traversal of attenuated macrophages^31,32^.

In contrast to these single gene studies, we here followed a more global approach by using comparative Illumina RNA-seq to identify differentially expressed host (*Bos taurus)* and parasite (*T. annulata*) genes between virulent and attenuated *T. annulata*-transformed leukocytes that could explain the attenuated phenotype of long-term passaged cells. In addition, we sought to identify genes that could be used as transcriptionally measurable candidate biomarkers for attenuation, with could eventually lead to a reduction in the number of required in vivo assessments for attenuation.

## Results

### Read mapping statistics

We applied a deep sequencing strategy to generate host and parasite transcript abundance profiles by comparing bovine leukocytes infected with virulent and attenuated passages of the Beja *T. annulata* strain from Tunisia. The host and parasite reads retained after sequential rounds of quality filtering were mapped to the ensemble* B. taurus* ARS-UCD1.2 and the *T. annulata* Ankara genome, respectively. The read mapping statistics are summarized in supplementary Fig. 1.

**Figure 1:**
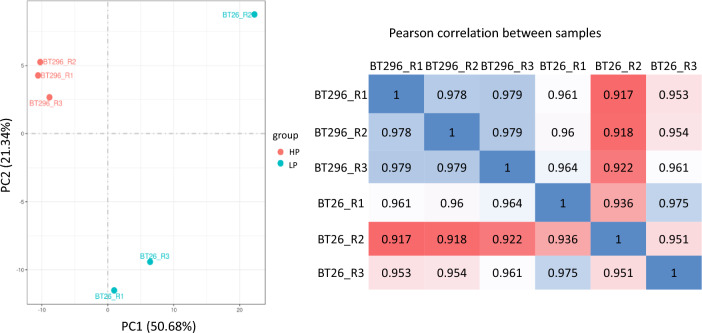
Principal component analysis (PCA) plots and inter-sample correlation heat map (R^2^: Square of Pearson correlation coefficient(R)) showing the clustering and the correlation between the samples (BT296 is the attenuated and BT26 is the virulent).

A demonstration that the data set represented differences in gene expression profiles between virulent and attenuated infected cells was provided by a Principal Component Analysis (PCA). As shown in Fig. 1, the PCA explains the clustering according to the passage level (low and high passage), with Principal Component 1 (PC1) represent 50.6% of most variation in the data and PC2 represent 21.3% of the second variation in the data. The correlation coefficient of samples between groups is calculated and drawn as heat maps which shows a Pearson correlation R^2^ greater than 0.9, confirming a high similarity between the samples.

### Global gene expression changes

#### *Global gene expression changes following *in vitro* serial propagation T. annulata infected leukocytes*

**Figure 2:**
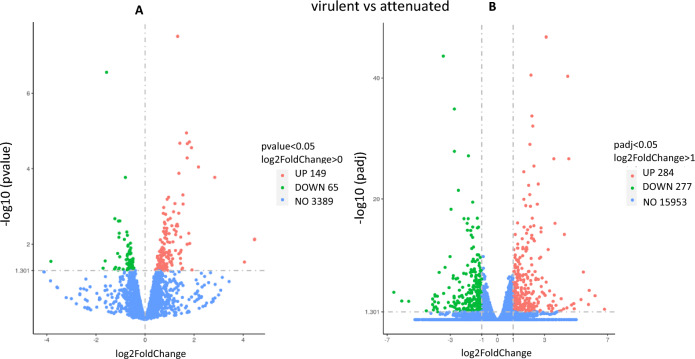
Differential Gene expression Volcano Map showing the up- and down-regulated genes as well as the unchanged genes in the parasite (**A**) and in the host (**B**). The abscissa in the figure is log2 Fold Change, and the ordinate is − log10 * p* adj or − log10 * p* value, the dashed line indicates the threshold line for differential gene screening criteria.

In total, 3538 parasite transcripts were detected across the six replicates sequenced (three from each passage), 149 of which were significantly up-regulated and 65 were down-regulated in the attenuated passage (Fig. 2A). Analysis of the bovine cell transcriptome revealed a total of 16,514 genes, 284 of which were significantly up-regulated, 277 were down-regulated and 15,953 genes were unchanged after attenuation (Fig. 2B).

#### Broad functional categories represented by the genes that significantly changed expression in the host and the parasite

**Figure 3:**
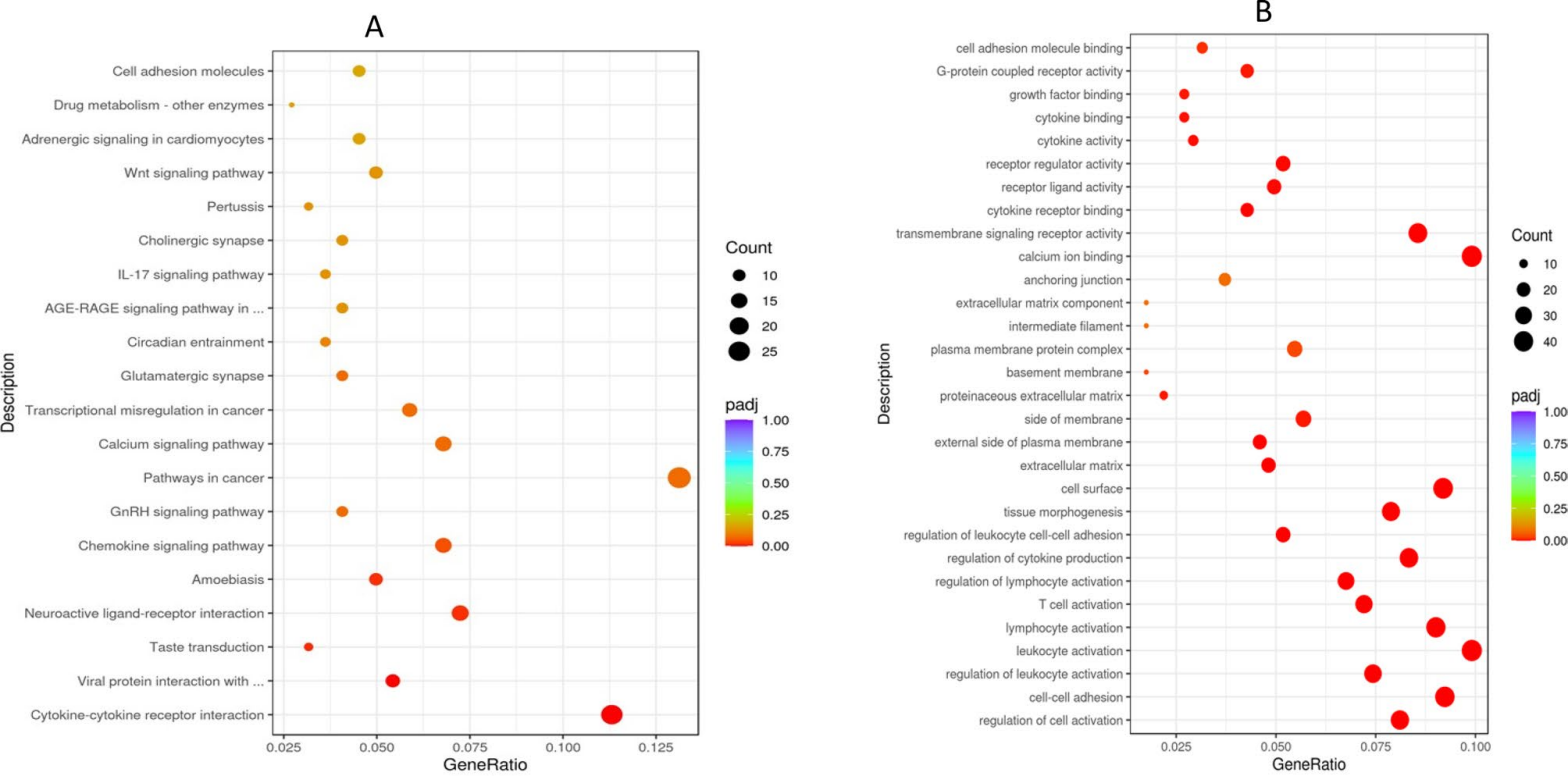
KEGG (**A**) and GO (**B**) enrichment GO enrichment analysis scatter plot of differentially expressed host genes. The abscissa in the graph (A) is the ratio of the number of differential host genes on the KEGG pathway to the total number of differential genes, and the ordinate is KEGG pathway. The abscissa in the graph (B) is the ratio of the differential gene number to the total number of differential genes on the GO Term, and the ordinate is GO Term.

The gene functions are classified into three groups including biological processes (BP), cellular components (CC) and molecular function (MF). The refined list of 171 differentially expressed bovine genes that exhibited a twofold or greater significant changes between virulent and attenuated cell lines were associated with the cell surface and with functions such as tissue morphogenesis, regulation of leukocyte cell–cell adhesion, regulation of cell adhesion and regulation of leucocyte and lymphocyte cell activation (Fig. 3).

Although there was no significant GO enrichment, the differentially expressed parasite genes were associated with different metabolic pathways such as protein folding, lipid biosynthetic process, cellular lipid metabolic process, chromosome organization, generation of precursor metabolites and energy (Supplementary Fig. 2b). KEGG analysis similarly identified parasite genes enriched in metabolic pathways such as carbon metabolism, oxidative phosphorylation, protein processing in the endoplasmic reticulum and phagosome and biosynthesis of secondary metabolites (Supplementary Fig. 2a).

### Transcriptional changes in *T. annulata-*infected bovine leukocytes during long-term in vitro culture

Because the loss of virulence and clinical pathogenicity of high passaged (attenuated) *T. annulata* transformed cells could directly be due to changes in host cell gene expression during the long-term process of in vitro passaging^20,24^, the bovine transcriptome of leukocytes of low and high passage were compared resulting in a total of 561 host genes that were significantly differentially expressed (284 were up- and 277 were downregulated). Based on the padj value, 168 genes were found with a fold change value greater than 2 with significant *p* adj values (< 0.05). The most up-regulated genes in high passage attenuated Beja cells were UPK3BL1 (*Bos taurus* uroplakin 3B-like) and P2RX3 (purinergic receptor), while the most down-regulated genes were ZNF618 (zinc finger protein 618) and the *B. taurus* CD69 molecule. A list of top 20 up and top 20 down-regulated genes is shown in Table 1.

**Table 1:** Top 40 up- and down-regulated host genes in attenuated passage based on the log2FoldChange and padj value.

Gene_id	Gene_name	Gene_description	Attenuated (average RPKM*)	Virulent (Average RPKM*)	log2FoldChange	padj
ENSBTAG00000020524	UPK3BL1	Bos taurus uroplakin 3B-like (UPK3BL1), mRNA. [Source:RefSeq mRNA;Acc:NM_001168011]	25.5484371	0.3468303	6.20492255	0.00010761
ENSBTAG00000000376	P2RX3	purinergic receptor P2X 3 [Source:VGNC Symbol;Acc:VGNC:32519]	37.0572054	0.6936606	5.83671086	1.40E-05
ENSBTAG00000003030	KCNQ2	potassium voltage-gated channel subfamily Q member 2 [Source:VGNC Symbol;Acc:VGNC:30487]	9.85363751	0	5.79271357	0.003043
ENSBTAG00000006806	KRT17	keratin 17 [Source:VGNC Symbol;Acc:VGNC:50398]	56.5290977	1.22118738	5.46777552	1.27E-08
ENSBTAG00000012630	PAMR1	peptidase domain containing associated with muscle regeneration 1 [Source:VGNC Symbol;Acc:VGNC:32562]	10.1895288	0.30529685	4.87936486	0.01556863
ENSBTAG00000018395	KIAA1217	KIAA1217 [Source:VGNC Symbol;Acc:VGNC:30566]	18.4306952	0.61059369	4.85316467	0.00212362
ENSBTAG00000048591	THBD	thrombomodulin [Source:HGNC Symbol;Acc:HGNC:11784]	16.6750819	0.61059369	4.71007582	0.00360285
ENSBTAG00000008944	CRB1	Bos taurus crumbs 1, cell polarity complex component (CRB1), mRNA. [Source:RefSeq mRNA;Acc:NM_001192482]	171.441218	7.37485138	4.53543835	2.45E-27
ENSBTAG00000002661	ISLR2	immunoglobulin superfamily containing leucine rich repeat 2 [Source:VGNC Symbol;Acc:VGNC:30299]	284.796605	12.7310177	4.47289549	5.13E-41
ENSBTAG00000004261	SPON2	spondin 2 [Source:VGNC Symbol;Acc:VGNC:35228]	98.5821353	5.22738488	4.25252666	7.42E-15
ENSBTAG00000003777	TIE1	Bos taurus tyrosine kinase with immunoglobulin like and EGF like domains 1 (TIE1), mRNA. [Source:RefSeq mRNA;Acc:NM_173965]	11.9451421	0.65212714	4.2094648	0.01658889
ENSBTAG00000005404	MSC	musculin [Source:VGNC Symbol;Acc:VGNC:31691]	26.5104467	1.57839235	4.05093325	0.00034497
ENSBTAG00000050719	−	calcium-dependent phospholipase A2 PLA2G2D1 [Source:NCBI gene;Acc:494318]	10.864386	0.6936606	4.04658787	0.03146184
ENSBTAG00000054512	IGSF1	Bos taurus immunoglobulin superfamily member 1 (IGSF1), mRNA. [Source:RefSeq mRNA;Acc:NM_001105048]	9.91758328	0.66250877	3.93311066	0.03703225
ENSBTAG00000025634	FMN1	formin 1 [Source:HGNC Symbol;Acc:HGNC:3768]	17.0322943	1.30425429	3.72041477	0.00404415
ENSBTAG00000008832	CCL1	C–C motif chemokine ligand 1 [Source:VGNC Symbol;Acc:VGNC:26943]	114.442319	9.05707389	3.66458396	1.12E-16
ENSBTAG00000007421	CDH5	cadherin 5 [Source:NCBI gene;Acc:414735]	31.246492	2.52544167	3.61207206	8.13E-05
ENSBTAG00000037553	KRT33A	Bos taurus keratin 33A (KRT33A), mRNA. [Source:RefSeq mRNA;Acc:NM_001099099]	227.067215	18.905438	3.58304894	2.45E-27
ENSBTAG00000017060	ITGB2	integrin subunit beta 2 [Source:VGNC Symbol;Acc:VGNC:30327]	22.5558378	1.94599286	3.54144292	0.00091222
ENSBTAG00000002739	PDE1C	phosphodiesterase 1C [Source:VGNC Symbol;Acc:VGNC:32673]	15.0138893	1.34578774	− 3.52040797	0.01313049
ENSBTAG00000006686	NPNT	nephronectin [Source:VGNC Symbol;Acc:VGNC:32209]	6.81535641	68.6539285	− 3.3327037	2.22E-09
ENSBTAG00000042166	RF00410	−	1.68557075	17.2356305	− 3.341479	0.01078732
ENSBTAG00000006855	TDRD1	tudor domain containing 1 [Source:VGNC Symbol;Acc:VGNC:35715]	3.05655381	32.6042682	− 3.4157894	4.53E − 05
ENSBTAG00000003398	KCNG1	potassium voltage-gated channel modifier subfamily G member 1 [Source:VGNC Symbol;Acc:VGNC:30442]	61.5126929	666.608932	− 3.4385742	2.36E-44
ENSBTAG00000005370	TMTC1	Bos taurus transmembrane and tetratricopeptide repeat containing 1 (TMTC1), mRNA. [Source:RefSeq mRNA;Acc:NM_001192761]	4.40013653	57.4349605	− 3.7038803	1.50E-08
ENSBTAG00000042696	RF00071	small nucleolar RNA, C/D box 73A [Source:HGNC Symbol;Acc:HGNC:10235]	2.38781081	32.1493473	− 3.7487039	0.00147441
ENSBTAG00000005425	GATA4	GATA binding protein 4 [Source:VGNC Symbol;Acc:VGNC:29268]	0.67787935	9.54307417	− 3.8148273	0.04964
ENSBTAG00000053497	GNLY	granulysin [Source:NCBI gene;Acc:404173]	2.04887114	28.7995165	− 3.8210854	0.00013213
ENSBTAG00000009341	CCDC146	Bos taurus coiled-coil domain containing 146 (CCDC146), mRNA. [Source:RefSeq mRNA;Acc:NM_001205487]	1.01377939	14.6271358	− 3.8396071	0.0153719
ENSBTAG00000009302	RCAN2	Bos taurus regulator of calcineurin 2 (RCAN2), transcript variant 2, mRNA. [Source:RefSeq mRNA;Acc:NM_001015632]	1.01073103	15.3083605	− 3.9136064	0.00772622
ENSBTAG00000046107	TTBK1	tau tubulin kinase 1 [Source:HGNC Symbol;Acc:HGNC:19140]	1.69165875	26.4921376	− 3.965986	0.00012693
ENSBTAG00000010111	TCF7L1	transcription factor 7 like 1 [Source:NCBI gene;Acc:515303]	2.06104713	32.20969	− 3.9795809	7.21E-05
ENSBTAG00000012368	IL21	interleukin 21 [Source:VGNC Symbol;Acc:VGNC:30141]	1.34358271	21.6281861	− 3.9958212	0.00108001
ENSBTAG00000024675	CYSLTR1	Bos taurus cysteinyl leukotriene receptor 1 (CYSLTR1), mRNA. [Source:RefSeq mRNA;Acc:NM_001099726]	0.99855504	16.4111412	− 4.0180996	0.00870421
ENSBTAG00000001068	ZCWPW1	zinc finger CW-type and PWWP domain containing 1 [Source:VGNC Symbol;Acc:VGNC:37125]	0.68701571	11.4433525	− 4.0661589	0.02374012
ENSBTAG00000048565	BASP1	Bos taurus brain abundant membrane attached signal protein 1 (BASP1), mRNA. [Source:RefSeq mRNA;Acc:NM_174780]	1.36185543	23.6780022	− 4.120178	0.00037734
ENSBTAG00000015094	VNN1	Bos taurus vanin 1 (VNN1), mRNA. [Source:RefSeq mRNA;Acc:NM_001024556]	1.02900375	17.8296211	− 4.1234276	0.00314342
ENSBTAG00000007589	SMAD9	Bos taurus SMAD family member 9 (SMAD9), mRNA. [Source:RefSeq mRNA;Acc:NM_001076928]	0.33285168	8.39456512	− 4.5214841	0.03301325
ENSBTAG00000003791	LPAR3	Bos taurus lysophosphatidic acid receptor 3 (LPAR3), mRNA. [Source:RefSeq mRNA;Acc:NM_001192741]	0.34807603	18.2533247	− 5.6437392	0.0009192
ENSBTAG00000002135	CD69	Bos taurus CD69 molecule (CD69), mRNA. [Source:RefSeq mRNA;Acc:NM_174014]	0	12.6541583	− 6.0743754	0.00080407
ENSBTAG00000025659	ZNF618	zinc finger protein 618 [Source:VGNC Symbol;Acc:VGNC:37314]	0.33893968	34.9199228	− 6.5764645	2.81E-05

*RPKM = reads Per Kilobase of transcript, per Million mapped reads.

**Figure 4:**
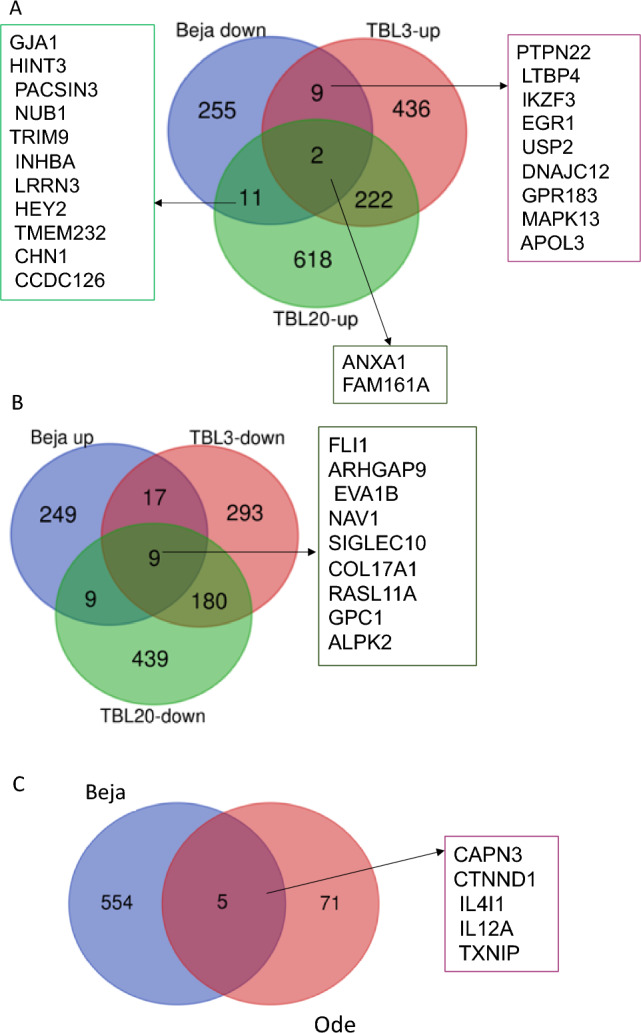
Venn diagrams showing the genes inversely differentially expressed in Beja high passage (attenuated) compared to TBL3, TBL20 and attenuated Ode. Panel A showing an inverse comparison of genes down-regulated in attenuated Beja vs genes up-regulated by infection; Panel B showing genes up-regulated in Beja vs genes down-regulated in TBL and panel C showing the common genes in attenuated passage between Beja and Ode cell lines.

We were wondering if our list of DEGs contain genes that are directly controlled by *T. annulata* infection and are down-regulated during the attenuation process. To identify this group of genes we merged the lists of genes upregulated upon infection with those downregulated in attenuated Beja. The list of genes significantly de-regulated by *T. annulata* between non-infected immortalized bovine B lymphocyte lines BL3 and BL20 compared to parasite infected TBL3 and TBL20 was previously published^33^. A comparison of our results with that study revealed that 11 and 13 genes were common with TBL3 and TBL20, respectively, whereas only two genes (ANXA and FAM161A) were common among the three datasets (Fig. 4, panel a). The other category of potentially interesting genes was those that were repressed by *T. annulata* infection (down-regulated in TBL3/TBL20) and upregulated in attenuated Beja. This category likely represents tumour suppressors and were more than pro-tumorigenic genes (only two genes) shared between TBL3, TBL20 and attenuated Beja (i.e., nine genes) (Fig. 4, panel b).

To date, comparative differential host cell gene expression of only one attenuated *T. annulata* (Anand, India) transformed macrophage, termed Ode has been reported^33,34^. For the purpose of finding common differentially expressed host genes between the Beja cell line studied herein and Ode that might be useful as attenuation biomarkers, a comparison of the differential transcriptome between virulent and attenuated passage of the two cell lines was conducted. The comparison showed only five common genes, three of which were up-regulated (CAPN3, CTNND1, IL4I1) and two were down-regulated in attenuated Beja passage (IL12A and TXNIP) (Fig. 4, panel C).

Activator protein 1 (AP-1) is a transcription factor whose constitutive activity is essential for *T. annulata*-induced leukocyte transformation^10^. Down-regulated AP-1 activity has been demonstrated in Indian (Ode) and Tunisian (Jed4) cell lines ^10,27,28^. We conducted a bioinformatic screen (PROMO) of our DEG list for the presence of potential AP-1 binding sites on their promoters (1000 base pairs upstream of the start codon). Among the 277 down-regulated genes and 284 up-regulated genes, a total of 30 and 39 genes possesses potential binding sites for AP-1 transcription factor, respectively (Supplementary Table 2).

Nuclear factor (NF-κB) is known to be regulator of *Theileria* transformed cell proliferation and survival^35^. In our DEGs, putative binding sites of NF-κB were found in the promoters of 13 up-regulated genes and 14 down-regulated genes (Supplementary Table 2).

### Transcriptional differences in *T. annulata* parasite between virulent and attenuated passage of Beja strain

As it has been demonstrated that virulence and attenuation of *T. annulata* transformed leukocytes is a parasite encoded trait^25,26^, the most interesting genes are those that were down-regulated during attenuation especially the ones with a signal peptide (SP) or transmembrane domain(s) (TMDs). These are potential virulence factors as they are likely to be secreted and transferred to either schizont membrane, host cytoplasm or host cell nucleus. These sets of genes hold the potential to interact with host cell signalling pathways that are associated with virulence of the infected host cell. Based on the p value, we selected the top 40 genes which were significantly up- or down-regulated. Using PiroplasmaDB, we found that five of them possessed signal peptides and ten had one to nine TMDs which suggest their involvement in host parasite interaction (Table 2).

**Table 2:** Top 40 up-and down regulated genes in *Theileria annulata* virulent and attenuated strain based on *P* value.

Gene_name	Gene_description	Attenuated (average RPKM*)	Virulent (average RPKM*)	log2FoldChange	*p* value	Computed GO Functions	# TM Domains	SignalP Peptide
Tap370b08.q2ca38.02c	Cytochrome C oxidase subunit III (COX3 homologue), putative	491.304754	165.981616	1.56559474	2.78E-07	Cytochrome-c oxidase activity;obsolete heme-copper terminal oxidase activity	7	N/A
TA06810	Hypothetical protein, conserved	67.7844536	29.4781952	1.20130623	0.00209882	N/A	0	MYSNRNITVVLLLYITHFVHS
Tap370b08.q2ca38.03c	Cytochrome B, putative	339.412059	160.394615	1.08141211	0.00243271	Electron transfer activity;oxidoreductase activity	9	N/A
TA07960	Ubiquinol-cytochrome c reductase complex, subunit, putative	90.3826372	45.0169167	1.0055784	0.00241293	Ubiquinol-cytochrome-c reductase activity	0	N/A
EPrG00000717687	rRNA	3746.44801	2147.23358	0.80304429	0.00017134	N/A	0	
TA11950	hypothetical P-, Q-rich protein family protein, putative	1148.83556	685.63827	0.74465277	0.00475935	N/A	1	MINNIKYLIFVLIFRSCIFVASS
TA03075	Transcription initiation factor (TBP homologue), putative	216.967375	362.893299	− 0.7420673	0.00151448	DNA binding	0	N/A
TA15770	Hypothetical protein	120.081482	202.018472	− 0.7504735	0.00458963	nucleic acid binding;zinc ion Binding	0	N/A
TA09685	Hypothetical protein, conserved	355.15881	602.645021	− 0.7628442	0.00106305	Nucleic acid binding;protein binding	0	N/A
TA05280	Hypothetical protein	144.195729	249.042043	− 0.7883609	0.00372746	N/A	4	N/A
TA03875	RNA poly(A)-binding protein, putative	161.595485	280.590833	− 0.796081	0.00261815	Nucleic acid binding	1	N/A
TA15120	Chromatin assembly protein, putative	120.319726	211.979633	− 0.8170525	0.00287531	N/A	0	N/A
TA06515	Hypothetical protein, conserved	98.4987798	176.816262	− 0.8440732	0.00160673	N/A	0	N/A
TA18745	Nicotinate-nucleotide adenylyltransferase-like protein, putative	70.273125	129.167696	− 0.8782003	0.00450453	Catalytic activity;nucleotidyltransferase activity	0	N/A
TA13830	Hypothetical protein	53.9241059	99.6059114	− 0.885301	0.00377777	N/A	0	N/A
TA03785	Proteasome subnit, putative	141.085401	264.287756	− 0.9055409	0.00064132	Endopeptidase activity;threonine-type endopeptidase activity	0	N/A
TA08715	Bacterial histone-like protein, putative	192.899489	368.459133	− 0.9336553	0.00225835	DNA binding	0	MFTYTNSFLLLIIIICLTVES
TA14000	Hypothetical protein, conserved	161.171375	313.652327	− 0.9605707	0.0005686	N/A	0	N/A
TA04835	Hypothetical protein, conserved	40.7638947	82.7821973	− 1.0220286	0.00206278	N/A	1	N/A
TA09825	Hypothetical protein, conserved	27.3304986	58.0526749	− 1.0868508	0.00322895	N/A	1	N/A
TA06365	Hypothetical protein	40.415858	91.4168075	− 1.177538	0.00137322	N/A	0	N/A
TA15565	Hypothetical protein, conserved	40.2554646	92.4333837	− 1.1992294	0.00083339	N/A	0	N/A
TA12655	Hypothetical protein	19.7224715	45.3836084	− 1.202331	0.00413025	N/A	0	N/A
TA03040	Hypothetical protein, conserved	20.6161283	49.5589351	− 1.2653718	0.00326846	Nucleic acid binding	0	N/A
TA02785	Hypothetical protein, conserved	17.277774	41.8650219	− 1.276828	0.00461403	N/A	0	MKIILILLIINFVIN
TA15135	Hypothetical protein, conserved	22.6745348	56.0820538	− 1.3064662	0.00295876	Protein binding	0	N/A
TA07115	40S Ribosomal S12-related protein, putative	41.1243984	102.839588	− 1.3223293	0.00330076	N/A	0	N/A
TA04840	60S Ribosomal L34 protein, putative	307.069625	769.990431	− 1.3262747	3.12E-08	Structural constituent of ribosome	0	N/A
TA04425	Ribosomal protein L18, putative	31.6375187	81.5085534	− 1.365315	0.00013327	N/A	0	N/A
TA08625	RNA polymerases I and III subunit (RPC19 homologue), putative	18.4423666	48.4360839	− 1.3930584	0.0015048	DNA binding;DNA-directed 5'-3' RNA polymerase activity;protein dimerization activity	0	N/A
TA07230	Hypothetical protein	46.3186425	124.313033	− 1.4243127	2.12E-05	N/A	1	MKIKILFIILIINFIKC
TA08610	Prefoldin subunit, putative	26.7160243	78.5055381	− 1.5550891	0.0004934	Unfolded protein binding	0	N/A
TA20245	Hypothetical protein	18.989486	55.907725	− 1.5578468	0.00086278	N/A	0	N/A
TA04760	Hypothetical protein, conserved	27.8035159	90.4935596	− 1.7025478	1.13E-05	Transcription coregulator activity	0	N/A
TA20225	Hypothetical protein	22.2882439	73.9714359	− 1.7306853	5.22E-05	N/A	1	N/A
TA09695	30S Ribosomal protein S8, putative	18.2494387	60.6542957	− 1.7327577	2.17E-05	Structural constituent of ribosome	0	N/A
TA13935	Hypothetical protein	21.4356829	76.061673	− 1.8271553	1.95E-05	N/A	0	N/A
TA15915	Hypothetical protein, conserved	20.0711006	73.5057958	− 1.8727383	2.79E-05	N/A	0	N/A
TA18140	Hypothetical protein	8.81402991	39.582718	− 2.166997	9.03E-05	N/A	0	N/A
TA08280	Hypothetical protein	3.22449848	24.2049842	− 2.9081575	0.00017088	N/A	2	N/A

*RPKM = reads Per Kilobase of transcript, per Million mapped reads.

We focused our analyses on the significantly down-regulated genes containing SPs or/and TMDs and looked at their orthologs in *T. parva* or *T. orientalis* to examine if their function is known. However, these genes were either not annotated (unspecified product or uncharacterized protein) or are known as integral membrane proteins (Supplementary Table 3). Only one gene (TA03875) was predicted to contain a NLS fragment (PKRKNIPGYNRRRTNNRT) located between position 116 and 133 aa (Supplementary Table 3).

Of the most significantly differentiated *Theileria* genes (based on the padj value), only three (TA04760, TA04840 and TA09695) were predicted to be involved in regulating different process such as gene expression, transcription, translation, biosynthesis, protein metabolic process and cellular metabolic process. It is interesting to note that these three parasite genes are hypothetical proteins that are associated with 29 common predicted pathways and are down-regulated in attenuated passages (Supplementary Fig. 3).

Using PiroplasmaDB, we looked at the orthologues of these genes in other Apicomplexa and their possible described function(s). TA04760 in *T. parva* is annotated as MED6 mediator sub complex component family protein and it regulates the transcription using polymerase II, thus its down-regulation following attenuation might affect expression of a subset of parasite genes that are potentially virulence factors. TA04840 and TA09695 are ribosomal proteins with similar functions and are involved in translation and structural constituent of the ribosome (Supplementary Table 4).

Furthermore, given the cancer-like phenotype induced by infection, we screened our dataset for genes that have a role in tumorigenesis. Interestingly, many of the transcripts that are differentially expressed (24 out of 40 DEGs) remain unannotated, despite publication of the *T. annulata* Ankara genome. As for the genes for which annotation is available, the roles of most of them in *T. annulata* pathogenesis or attenuation remain uncharacterised and only few are known to possess transcription coregulator activity (TA04760), nucleic acid and protein binding (TA09685, TA15135, TA03040) and nucleic acid and zinc ion binding (TA15770). All these genes are down-regulated in attenuated passage.

**Figure 5:**
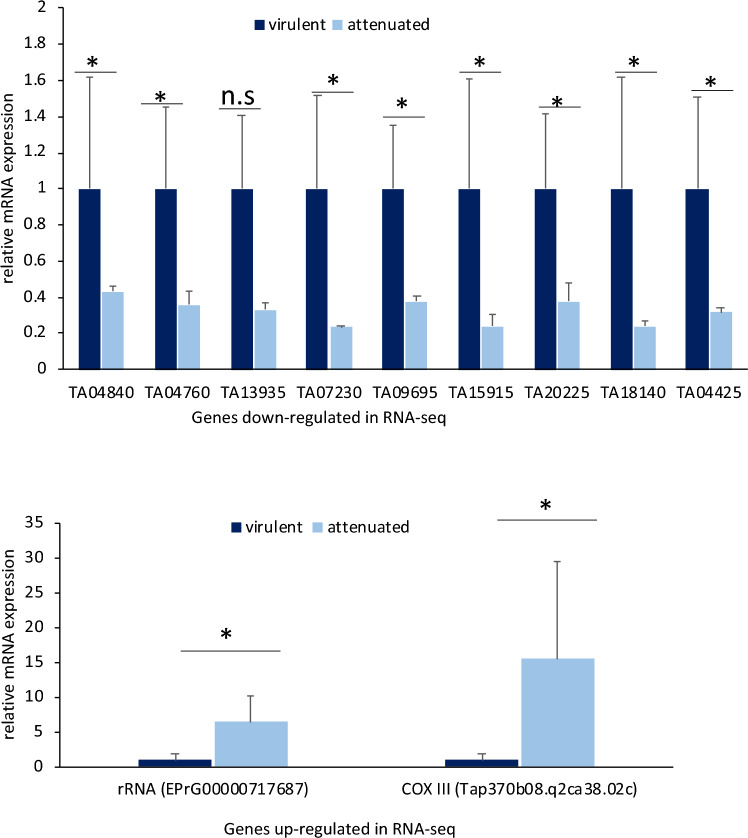
qRT-PCR validation of the selected up or down-regulated *Theileria* genes identified by RNA-seq. The y-axis shows the relative mRNA expression. *Theileria annulata* actin was used as a reference gene. Asterisks show significant difference in gene expression; (n.s) = non-significant.

For validation of Illumina RNA-seq determined levels of parasite gene expression by RT-qPCR, we selected 11 parasite genes which exhibited significant differential expression based on their padj values; nine of them were down regulated and two were up-regulated (supplementary Table 1). The comparative RNA seq results were corroborated by the qRT-PCR data (Fig. 5).

## Discussion

The mechanisms by which the multinucleated schizont stage of *T. annulata* hijacks host cell intracellular signalling and transcription pathways to reversibly transform infected leukocytes are not fully understood. Similarly, the complex interplay between *T. annulata* and the bovine macrophage during the generation of attenuated cell-line vaccines is not unravelled either. The amount of host and parasite genetic information is increasing exponentially and so are the resources with which to investigate the complex host–pathogen interactions. One such resource, RNA-seq analysis, is suited for investigating complex host–pathogen interactions as it allows for the simultaneous analysis of the expression of thousands of genes. Here, we report on an RNA-seq analysis comparing host and parasite mRNA levels in a virulent and attenuated *T. annulata* cell line from Tunisia.

The analysis identified over 500 bovine host genes and 200 parasite genes that exhibited a statistically significant differential expression, implying that there are differences in transcriptional pathways between virulent and attenuated cell lines. Overall, there was good agreement between the RNA-seq data and the qRT-PCR results for the genes exhibiting cell line-specific differential expression that were selected for validation. A demonstration that the data set represents differences in gene expression profiles between virulent and attenuated cell lines was provided by PCA where clustering of the samples with passage level can clearly be observed (Fig. 1), and PC1 explained 50.6% of variation in the data.

In order to include genes of putatively greater biological relevance, we refined the list of differentially expressed genes to include only those that exhibited a twofold or greater average difference in expression between the virulent and attenuated (Table 1). Based on the fold change value, the largest difference between virulent and attenuated cell line was observed for the host more than parasite genes. A total of 168 host genes showed a fold change value of 2 or more with significant padj values. Among these genes, 99 were up-regulated and 69 were down-regulated. Given the cancer-like transformation of infected macrophages by *T. annulata*, it is not surprising that a large proportion of the differentially expressed genes with fold change above the twofold cut-off, encode proteins that have been associated with tumours. The most striking host gene expression difference (exceeding sixfold) was observed for UPK3BL1 (*Bos taurus* uroplakin 3B-like) followed by P2RX3 (purinergic receptor), KCNQ2 (potassium voltage-gated channel subfamily Q member 2) and KRT17 (keratin 17) with a fold change of 5.8, 5.7 and 5.4, respectively.

UPK3BL1 is implicated in stabilizing and strengthening the urothelial cell layer of the bladder, it has been shown to be highly expressed in mesotheliomas and in the urothelial tumours of the urinary bladder^36^. P2RX3, has been reported to be involved in tumour formation and play an important role in malignant transformation of several different cell types and they are highly expressed in cancer^37,38^. The biological relevance of its high expression in attenuated *T. annulata* infected cell lines has not been investigated. KCNQ2 gene is known to encode for subunits of a potassium channel complex and to be involved in membrane polarisation, they are acting as a suppressors or activator of gastrointestinal cancer^39^. The most down-regulated gene was ZNF618 (zinc finger protein 618) followed by CD69 (*Bos taurus* CD69 molecule), LPAR3 (*Bos taurus* lysophosphatidic acid receptor 3) with a fold change of − 6.5, − 6 and − 5.6, respectively. CD69 is a glycoprotein type II known to regulate inflammation through T-cell migration and retention in tissues and has a crucial role in inducing the exhaustion of tumour-infiltrating T cells^40,41^. CD69 is also an AP-1 target gene^42,43^, its role in *T. annulata* pathogenesis and attenuation might be important to investigate. As already mentioned, the genes that exhibited statistically significant passage differences in expression and are also supported by the highest fold difference in expression can be investigated as candidates underlying attenuation.

A comparison of Beja DEGs with attenuated Ode showed five common genes. Two of them were down-regulated (IL12A and TXNIP), which could be potential attenuation markers, and three were up-regulated (CAPN3, CTNND1, IL4I1). The up-regulated genes could be that they are not involved in the attenuation process but rather other mechanisms such as immunosuppression or inflammation. For example, IL4I1 (interleukin 4 induced 1) protein may play a role in immune system escape as it is expressed in tumour-associated macrophages and it was described as a metabolic immune checkpoint which promotes tumour progression^44^. While Calpain-3 (CAPN3) is an intracellular cysteine protease, it has been shown in human melanoma that a variant of this gene called hMp84 increases the intracellular production of ROS (Reactive Oxygen Species) leading to DNA damage^45^ which has been also confirmed in *Theileria* to cause an oxidative stress by elevation of ROS and disruption of the redox balance which are required for parasite transformation^46^. This is interesting as it might explain why in attenuated macrophages H2O2 type of oxidative express accumulates in spite of lower JNK (an antioxidant) activity^47^. It should be noted that H2O2 levels in virulent and attenuated *T. annulata*-transfomed Ode macrophages inversely correlated with their Matrigel traversal capacity^47^.

Parasite-dependent induction of key host cell transcription factors (AP-1, NF-kB, etc.) in *Theileria annulata* transformed cells causes massive reprogramming of the host cell transcriptome^33,48^. Thousands of host cell genes increase or decrease upon *Theileria annulata*-induced cell transformation. We conducted an inversed comparison with previously published *T. annulata* infected BL3 and BL20 cell lines differential transcriptome in order to find genes that have been induced by infection and are down-regulated in attenuated passage or vice-versa (Fig. 3). For example, MAPK13 (mitogen-activated protein kinase 13) has been found in gynaecological cancer to be preferentially expressed in cancer stem cells and it is implicated in the tumour-initiation^49^. It was induced by infection in TBL20 but down-regulated in attenuated Beja (log2 Fold Change = − 1.2; *p* adj = 0.0001). AnxA1 (annexin A1) is a phospholipid-binding protein that has been described to have either activator or suppressor role in different cancer types and stages^50^. In TBL3 and 20, this gene was up-regulated by infection. It is found to be significantly down-regulated in attenuated transcript (log2 Fold Change = − 1.08; *p* adj < 0.001). FAM161A (centrosomal protein) is known to be involved in development of retinal progenitors during embryogenesis and it is a part of microtubule-organizing centres in cultured cells and it has been demonstrated to be involved in the intracellular microtubule network^51,52^, it was down-regulated in attenuated Beja (log2 Fold Change was − 1.005; *p* adj value = 0.01).

It has also been shown in breast cancer research that apolipoprotein modulates the cholesterol metabolism and stimulates the proliferation, migration, and tumour growth^53^. In our list Apolipoprotein 3 (APOL3) is induced by *Theileria* infection and down-regulated upon attenuation (log2 Fold Change was − 1.09; *p* adj value < 0.001) which suggest its implication in the attenuation process. Tumour protein 63 (p63) is known to enhance the migration, invasion, and tumour growth in human cancer^54^. This gene was down-regulated in attenuated passage (log2 Fold Change was − 1.09; *p* adj value < 0.001).

ARHGAP9 (Rho GTPase-activating protein 9), a tumour suppressing gene in bladder cancer^55^ that has been reported to be suppressed by infection in TBL, was significantly up-regulated in the attenuated passage (log2 Fold Change = 1.6; *p* adj < 0.001) but other ARHGAP were significantly down-regulated such as ARHGAP15 (log2 Fold Change = − 1.05; *p* adj < 0.001) and ARHGAP29 (log2 Fold Change = − 1.32; *p* adj < 0.001). These genes could also be evaluated as candidates involved in the attenuation process.

Our results suggest that long term passage of infected cells primarily results in transcriptional down-regulation of some cytokines (e.g., IL6, IL12 and IL21) and genes that may be active in pathways controlling the interaction between the cytokines and their receptors. Remodelling the host cell transcriptome in this way by attenuation appears consistent with the previous suggestion that upregulation of pro-inflammatory cytokines might play a role in pathology. This was on the basis of the observation that the clinical signs of *T. annulata* infection, particularly fever, cachexia, leucopoenia and anaemia are the same signs that follow experimental administration of pro-inflammatory cytokines^56^.

A high proportion of the differentially expressed genes (51 host genes in total where 14 of them had fold changes between 2 and 6) are unannotated and the importance of these proteins is unclear at this time. However, identification of these genes may provide vital information pertaining to attenuation of *T. annulata* infected cell lines. It is also important to mention that besides the reduced expression of pro-inflammatory cytokines that we confirm, attenuation has also previously been associated with reduced expression of the matrix metalloproteinase MMP9^25^, which is linked to the invasive capacity of virulent cell lines^57^. In *T. annulata*-transformed macrophages, MMP9 transcription is regulated by Activator protein 1 (AP-1)^27^. Down-regulated AP-1 activity has been demonstrated in Indian (Ode) and Tunisian (Jed4) cell lines^10,27,28^. However, our comparative RNA-seq transcription profiles did not reveal a downregulation of this candidate attenuation marker. Interestingly, MMP9 had very low level of read counts in our RNA-seq data. Therefore, differential expression of genes other than MMP9 could be associated with diminished virulence of attenuated Beja, such as those presented in Fig. 4 (for example MAPK13, ANXA, etc.…). Also, the fact that Beja cell line is less virulent than Jed4 at low passage should be taken into account as it might be that the expression level of AP-1 is low at virulent passage^58^. Although we did not measure AP-1 activity in virulent versus attenuated Beja, we found potential AP-1 binding sites on promoters of 30 genes that were down-regulated in attenuated Beja. The presence of potential AP-1 binding sites on down-regulated gene promoters and dampened AP-1 activity in attenuated cells suggest that reduced AP-1 activity might explain the attenuated phenotype of high passage Beja leukocytes. It has also been reported that NF-κB (nuclear factor kappaB) is activated in *Theileria* transformed cells via the IKK (IκB kinase) complex to regulate cell proliferation and protect it against apoptosis^35,59^ but its role in the attenuation process is not yet demonstrated. In our DEGs list we found NF-κB binding sites in 13 up-regulated genes and 14 down-regulated genes promoters. The role of NF-κB in the attenuation process of *T. annulata* need to be investigated.

As regards to the parasite, it has previously been suggested that both altered gene expression and clonal selection of parasite populations may be involved in the loss of pathogenicity of *T. annulata* during continuous in vitro culture^60^. For the parasite transcript, only five down-regulated genes had a fold change more than − 2, all of which were hypothetical proteins. Availability of Beja genome sequence will likely make it possible to expand the annotation available for these genes, particularly given that analysis of antigen gene sequences, including TaSP, have shown that the Tunisian *T. annulata* differs from parasites from other locations including Turkey from where the reference genome is derived^61^. There was one up-regulated parasite gene that had a fold change of 3 (cytochrome C oxidase subunit III), while the most of the DEGs have a fold change value less than 2. Nonetheless, we below describe some of the parasite genes such as TA08610, TA03875, TA18945, TA20090 and TA09695 that likely have a role in *T. annulata* virulence.

TA08610 is a gene coding for the prefoldin subunit (one of the prefoldin complex), a cytoplasmic chaperone involved in protein folding with multiple roles in different tumours. The alpha subunit has a tumour-suppressing role and the beta subunit is involved in tumorigenesis^62,63^. This gene was significantly down-regulated in the attenuated cell line dataset (log2 Fold Change = − 1.5; *p* < 0.001). TA03875 codes for RNA poly(A)-binding protein which is a post-transcriptional regulator of gene expression controlling mRNA stability, polyadenylation, and other functions. An aberrant expression of this gene has previously been reported to be associated with the development of breast cancer^64,65^, this gene was down-regulated in attenuated passage in the attenuated Beja passage (log2 Fold Change = − 0.9; *p* < 0.001). We also found that this gene contains a nucleus localization signal (NLS) which suggest its possible transport into the host nucleus, however this needs to be confirmed since previous studies showed that some genes such as TaMISHIP *(T. annulata* proline‐rich microtubule and SH3 domain‐interacting protein) presented NLS but they were not detected in the host nucleus^66^. Additionally, we checked some of the previously characterised *T. annulata* effector proteins (TaSP, TashATs, TaHSP90, TaPIN1, Ta-p104; reviewed by Tajeri and Langsley (2021)^67^ for their possible differential expression, all of these genes were unchanged in our list.

It is known that protozoa execute a strict regulation on the expression of genes that are involved in their pathogenicity^68^. In *Theileria* transcripts, we found three hypothetical proteins (TA04840, TA04760 and TA09695) that have been reported to be involved in gene expression and in translation. TA04760 is involved in the regulation of various process such as cellular process, biological process, transcription process, regulation of gene expression and RNA biosynthetic processes. These genes were down-regulated in the attenuated passage. The role of the mentioned hypothetical proteins needs to be characterized in order to confirm their implication in the attenuation process and if they are involved in regulation of the host gene expression. If this is confirmed, it might be helpful for the control of the *T. annulata* by manipulating gene expression as it was described in *Plasmodium falciparum* by using Peptide Nucleic Acids (PNAs) for gene silencing which induced down-regulation of stably expressed transgene and endogenous essential gene led to reduction of plasmodium viability^69^.

In conclusion, our comparative analysis catalogued for the first time differentially expressed host and parasite genes between virulent and attenuated Tunisian cell lines (Beja). A major finding of the study is that both the bovine host and *Theileria* display changes in gene expression following attenuation. Besides the use of statistical significance and average fold change to prioritize candidates, recent advances in cancer also enabled description of additional genes with known roles in different tumour and their potential role in *T. annulata* pathogenesis. In particular, the number of host (e.g., ZNF618, LPAR3, APOL3, CD69) and parasite (TA03875, TA04840, TA04760 and TA09695) differentially expressed genes listed herein could be further validated using previously described methods such as the Matrigel chamber assay^33^, Enzyme-Linked Immunosorbent Assay (ELISA)^70^, immunoblotting and immunofluorescence^71^, in order to find potential attenuation markers. Such studies would contribute to a better understanding of the mechanism driving attenuation, reduce the time needed to establish an attenuated cell line and contribute to the application of 3Rs principle in *Theileria* research by reducing the number of animals required for the validation of attenuation.

## Materials and methods

### Theileria annulata cell line and culture

The *T. annulata*-infected cell line used in this study is a stock of the Beja strain (also referred to as CL1 in literature). The strain was originally isolated in 1989 from an infected five-year-old crossbred cow with an acute form of TT and it was used to vaccinate cattle in Tunisia against tropical theileriosis^24,72^. Phenotyping of the Beja strain, up to passage 300 using flow cytometry and specific monoclonal antibodies, revealed a dominance of the myeloid lineage (macrophages/monocytes). Furthermore, the markers associated to B cells were not entirely absent relative to the parental lines, which tend to indicate that a minor population of infected B cells (2–8%) may persist during the process of attenuation^58^. The cell line was frozen in liquid nitrogen and was resuscitated and propagated in RPMI supplemented with 10% FBS (RPMI-FBS), 2 mM L-alanyl-l-glutamine, 100 IU/ml penicillin and 100 μg/ml streptomycin. The culture flasks (three replicates for each passage) were incubated at 37 °C in a 5% CO_2_-in-air atmosphere.

### RNA extraction

RNA was extracted from three replicates of both virulent (passage 26) and attenuated (passage 296) cells using a Direct-Zol RNA miniprep plus kit (Zymo Research Europe GmbH, Freiburg, Germany) according to the manufacturer’s guidelines. The transcriptome of the *T. annulata* virulent and attenuated Beja cell line from Tunisia was subsequently determined using Illumina RNA sequencing.

### RNA quantification, qualification and library preparation for transcriptome sequencing

Three purified RNA replicates from each passage with concentrations from 168 to 361 ng/µl were sequenced at Novogene laboratories (Cambridge Science Park, Cambridge, CB4 0FW, United Kingdom). Messenger RNA was purified from total RNA using poly-T oligo-attached magnetic beads. After fragmentation, the first strand cDNA was synthesized using random hexamer primers, followed by second strand cDNA synthesis using dUTP for directional library or dTTP for non-directional library. The library was checked with Qubit and real-time PCR for quantification and bioanalyzer for size distribution detection. Quantified libraries were pooled and sequenced on Illumina platforms. The clustering of index-coded samples was performed on a cBot Cluster Generation System using TruSeq PE Cluster Kit v3-cBot-HS (Illumina) according to the manufacturer’s instructions. After cluster generation, the library preparations were sequenced on an Illumina Novaseq platform and 150 bp paired-end reads were generated. Raw data were first processed through in-house perl scripts where clean reads were obtained by removing reads containing adapter, reads containing poly-N and low-quality reads. The RNA seq raw data has been submitted to the SRA database (sequence read archive) under the accession number PRJNA957332.

### Mapping to the reference genome and quantification of gene expression levels

Reference genome annotation files were downloaded for the *T. annulata* Ankara cell line (PRJNA16308) and *Bos taurus* (PRJNA391427). Index of the reference genome was built using Hisat2 v2.0.5 and paired-end clean reads were aligned to the reference genome using Hisat2 v2.0.5. The mapped reads of each sample were assembled by StringTie (v1.3.3b)^73^ in a reference-based approach. Feature Counts v1.5.0-p3 was used to count the reads numbers mapped to each gene after which the FPKM (number of Fragments Per Kilobase of transcript sequence per Millions base pairs sequenced) of each gene was calculated based on the length of the gene and reads count mapped to this gene.

### Differential expression analysis, GO and KEGG enrichment analysis of differentially expressed genes

Differential expression analysis of the two groups was performed using the DESeq2 R package (1.20.0). The resulting *P*-values were adjusted using the Benjamini and Hochberg’s approach for controlling the false discovery rate^74^. In general, genes with *p* value ≤ 0.05 were assigned as differentially expressed. A more stringent criteria of an absolute fold change of 2 or more with *p* adj value ≤ 0.05 was initially applied to both the host and parasite genes. The list of differentially expressed parasite genes was subsequently extended to include genes that exhibited significant differential expression based on the *p* value only, but additional possessed important features such as transmembrane domains and/or signal peptides.

Gene Ontology (GO) enrichment analysis of differentially expressed genes was implemented by the clusterProfiler R package, in which gene length bias was corrected. GO terms with corrected* p*-values less than 0.05 were considered significantly enriched by differentially expressed genes. Cluster Profiler R package was used to test the statistical enrichment of differential expression genes in KEGG (Kyoto Encyclopedia of Genes and Genomes) pathways^75^.

### Additional bioinformatic screen: in silico search for differentially expressed *T. annulata* proteins with signal peptides, transmembrane domains or nuclear localization signals and prediction of AP-1 binding sites on promoters from differentially expressed bovine genes

PiroplasmaDB (https://piroplasmadb.org/piro/app/) was used to further characterize *Theileria* DEGs including their assignment to GO functions and predictions of the presence of a signal peptide (SP) and transmembrane domains (TMDs). In order to find nuclear localization signals (NLS) we used the novopro lab online tool (https://www.novoprolabs.com/tools/nls-signal-prediction). We also conducted a bioinformatic screen of our host DEGs list for the presence of AP-1 (activator protein 1) and NF-kappa B potential binding sites using the PROMO website (http://alggen.lsi.upc.es/cgi-bin/promo_v3/promo/promoinit.cgi?dirDB=TF_8.3). A promoter region was considered 1000 bp upstream of the start codon and retrieved from Ensembl website (https://www.ensembl.org/biomart/martview/151e6b41a7a359e264c5aa1d96328fa7).

### Validation of RNA-seq results by qRT-PCR

The differential gene expression was validated by quantitative reverse transcription PCR (qRT-PCR). cDNA synthesis was conducted using the ProtoScript® II First Strand cDNA Synthesis Kit (New England BioLabs Inc, Ipswich, US) according to manufacturer´s instructions from the same RNA samples used for the RNA-seq. The specific primer pairs for 11 differentially expressed *T. annulata* genes were designed using Geneious software^76^ (Supplementary Table 1). The *Theileria* actin gene (TA13410) was used as a reference gene. The qPCR reactions were performed using Luna® Universal qPCR Master Mix (New England BioLabs Inc) according to the manufacturer’s protocol. The 2^−ΔΔCT^ methodology was used to estimate the relative gene expression levels^77^.

### Supplementary Information

Supplementary Information.

## Data Availability

RNA seq raw reads have been uploaded to Sequence Read Archive (SRA) repository under the BioProject Number PRJNA957332.
