# Supplementary material for: Dual RNA-seq to catalogue host and parasite gene expression changes associated with virulence of T. annulata-transformed bovine leukocytes: towards identification of attenuation biomarkers
Source: Sci Rep. 2023 Oct 24;13:18202. doi: 10.1038/s41598-023-45458-9 (PMC10598219; doi:10.1038/s41598-023-45458-9)
Supplement: Supplementary file 1 — Supplementary Information. [file 41598_2023_45458_MOESM1_ESM.docx]

**Supplementary table 1.** **Details of the quantitative RT-PCR primers used and designed in this study**

| Gene Id/description | Sequence name & direction | Product size (bp) | Sequence (5’-3’) |
| --- | --- | --- | --- |
| EPrG00000717687 (rRNA) | EPr687-F | 150 | AGTGACGCGCATGAATGGAT |
|  | EPr687-R |  | TCAAGTCGTTTCACAAAGCCGG |
| ribosomal protein L18, putative | TA04425-F | 159 | ACACATGATGTACTTTGTTTTGTTTCT |
|  | TA04425-R |  | TGCTGCTTTTATTGTACCACCT |
| hypothetical protein, conserved | TA04760-F | 150 | CAAGGATCAATTTTTATGTGTCCACCA |
|  | TA04760-R |  | TGGTTTCGGATTCCAATCATATCCA |
| 60S ribosomal L34 protein, putative | TA04840-F | 149 | CTGGTGCTCGTCTTGTTCTTCA |
|  | TA04840-R |  | TGGTCTTGATACTGTACGATTTCTATG |
| hypothetical protein | TA07230-F | 150 | AGGAAGAATTAAAAACAACACCAATGA |
|  | TA07230-R |  | TGAGTTTGGTTTTAGTTTGTTGTGGA |
| 30S ribosomal protein S8, putative | TA09695-F | 150 | AAAACCTAGTCGTGATATTTGGGT |
|  | TA09695-R |  | GCAATGAGCATTTTACCACCAA |
| hypothetical protein | TA13935-F | 150 | TGGAGAATTTGAAGATTTGGGTGTG |
|  | TA13935-R |  | GTAGCAAATTTATAAAGTGCTCCTACA |
| hypothetical protein, conserved | TA15915-F | 150 | TGGTAGAGTATGGAAAGGAACATCA |
|  | TA15915-R |  | CCTTAGCTTCCTTTTTGATTTCTTCCA |
| hypothetical protein | TA18140-F | 184 | TGGATATTATTTGGAAGAAAAAGATG |
|  | TA18140-R |  | TTCACCCAAACTTTCAATATCA |
| hypothetical protein | TA20225-F | 183 | TGGTCAAATTGGTTAACATTTGAAGA |
|  | TA20225-R |  | ACAACTTGCTATACCTGCTGT |
| Tap370b08.q2ca38.02c (cytochrome C oxidase subunit III | Tap370-F | 150 | TCAAGGTGATGAATACTCATTGGT |
|  | Tap370-R |  | CCACAATTTTCAGAAGCAAAGGC |
| *T. annulata* actin ii, putative (TA13410), partial mRNA | TA13410-F | 150 | GACATTAAGGAGCGGTGCTG |
|  | TA13410-R |  | AGTAGTGCCGTCTGGGAGTTT |

**Supplementary table 2: Differentially expressed genes (DEGs) with a potential AP-1 (activator protein 1) and NFkB (nuclear factor kappaB) binding sites**

|  | **Gene stable ID** | **Gene description** | **Gene name** | **Status in attenuated Beja** |
| --- | --- | --- | --- | --- |
| **AP-1** | ENSBTAG00000000381 | sphingosine-1-phosphate receptor 4 [Source:VGNC Symbol;Acc:VGNC:34255] | S1PR4 | Up |
|  | ENSBTAG00000005313 | EPH receptor B3 [Source:VGNC Symbol;Acc:VGNC:28539] | EPHB3 | Up |
|  | ENSBTAG00000006232 | WD repeat domain 86 [Source:VGNC Symbol;Acc:VGNC:53682] | WDR86 | Up |
|  | ENSBTAG00000006806 | keratin 17 [Source:VGNC Symbol;Acc:VGNC:50398] | KRT17 | Up |
|  | ENSBTAG00000008331 | transmembrane protein 54 [Source:VGNC Symbol;Acc:VGNC:36097] | TMEM54 | Up |
|  | ENSBTAG00000008832 | C-C motif chemokine ligand 1 [Source:VGNC Symbol;Acc:VGNC:26943] | CCL1 | Up |
|  | ENSBTAG00000008868 | calpain 3 [Source:VGNC Symbol;Acc:VGNC:26747] | CAPN3 | Up |
|  | ENSBTAG00000010270 |  |  | Up |
|  | ENSBTAG00000010371 | ChaC glutathione specific gamma-glutamylcyclotransferase 1 [Source:VGNC Symbol;Acc:VGNC:27262] | CHAC1 | Up |
|  | ENSBTAG00000010416 | Ras and Rab interactor 3 [Source:VGNC Symbol;Acc:VGNC:53950] | RIN3 | Up |
|  | ENSBTAG00000012219 | chondroitin sulfate proteoglycan 4 [Source:VGNC Symbol;Acc:VGNC:57158] | CSPG4 | Up |
|  | ENSBTAG00000012682 | unc-13 homolog A [Source:VGNC Symbol;Acc:VGNC:36664] | UNC13A | Up |
|  | ENSBTAG00000012834 | arylsulfatase family member I [Source:VGNC Symbol;Acc:VGNC:26177] | ARSI | Up |
|  | ENSBTAG00000013736 | prominin 1 [Source:VGNC Symbol;Acc:VGNC:33364] | PROM1 | Up |
|  | ENSBTAG00000014358 | eva-1 homolog B [Source:VGNC Symbol;Acc:VGNC:52770] | EVA1B | Up |
|  | ENSBTAG00000014612 | dedicator of cytokinesis 2 [Source:VGNC Symbol;Acc:VGNC:28157] | DOCK2 | Up |
|  | ENSBTAG00000014855 | microtubule associated monooxygenase, calponin and LIM domain containing 2 [Source:VGNC Symbol;Acc:VGNC:31458] | MICAL2 | Up |
|  | ENSBTAG00000014915 | ETS variant transcription factor 5 [Source:VGNC Symbol;Acc:VGNC:28631] | ETV5 | Up |
|  | ENSBTAG00000016881 | alanyl aminopeptidase, membrane [Source:VGNC Symbol;Acc:VGNC:25960] | ANPEP | Up |
|  | ENSBTAG00000020674 | lymphotoxin beta [Source:VGNC Symbol;Acc:VGNC:31068] | LTB | Up |
|  | ENSBTAG00000023073 | family with sequence similarity 89 member A [Source:VGNC Symbol;Acc:VGNC:28842] | FAM89A | Up |
|  | ENSBTAG00000025634 | formin 1 [Source:HGNC Symbol;Acc:HGNC:3768] | FMN1 | Up |
|  | ENSBTAG00000038240 |  |  | Up |
|  | ENSBTAG00000048682 | zinc finger protein 548 [Source:NCBI gene (formerly Entrezgene);Acc:504913] | ZNF548 | Up |
|  | ENSBTAG00000050361 |  |  | Up |
|  | ENSBTAG00000050719 | calcium-dependent phospholipase A2 PLA2G2D1 [Source:NCBI gene (formerly Entrezgene);Acc:494318] | PLA2G2D1 | Up |
|  | ENSBTAG00000052473 |  |  | Up |
|  | ENSBTAG00000052516 | cytidine deaminase [Source:VGNC Symbol;Acc:VGNC:27055] | CDA | Up |
|  | ENSBTAG00000001839 | OCIA domain containing 2 [Source:VGNC Symbol;Acc:VGNC:32396] | OCIAD2 | Down |
|  | ENSBTAG00000004126 | myeloid leukemia factor 1 [Source:NCBI gene (formerly Entrezgene);Acc:533379] | MLF1 | Down |
|  | ENSBTAG00000004971 | GRAM domain containing 1C [Source:VGNC Symbol;Acc:VGNC:29624] | GRAMD1C | Down |
|  | ENSBTAG00000007388 | zinc finger CCCH-type containing 12D [Source:VGNC Symbol;Acc:VGNC:37100] | ZC3H12D | Down |
|  | ENSBTAG00000007704 | ELOVL fatty acid elongase 7 [Source:VGNC Symbol;Acc:VGNC:28453] | ELOVL7 | Down |
|  | ENSBTAG00000009080 | integrin subunit beta 6 [Source:VGNC Symbol;Acc:VGNC:30331] | ITGB6 | Down |
|  | ENSBTAG00000009294 | delta 4-desaturase, sphingolipid 2 [Source:VGNC Symbol;Acc:VGNC:27991] | DEGS2 | Down |
|  | ENSBTAG00000010103 | tripartite motif containing 9 [Source:VGNC Symbol;Acc:VGNC:36353] | TRIM9 | Down |
|  | ENSBTAG00000013010 | coiled-coil domain containing 126 [Source:VGNC Symbol;Acc:VGNC:26842] | CCDC126 | Down |
|  | ENSBTAG00000013284 | serum/glucocorticoid regulated kinase family, member 3 [Source:NCBI gene (formerly Entrezgene);Acc:504480] | SGK3 | Down |
|  | ENSBTAG00000014972 | prostaglandin E receptor 4 [Source:VGNC Symbol;Acc:VGNC:33504] | PTGER4 | Down |
|  | ENSBTAG00000018043 | lecithin-cholesterol acyltransferase [Source:VGNC Symbol;Acc:VGNC:30809] | LCAT | Down |
|  | ENSBTAG00000018059 | CD80 molecule [Source:VGNC Symbol;Acc:VGNC:27048] | CD80 | Down |
|  | ENSBTAG00000018463 | vimentin [Source:VGNC Symbol;Acc:VGNC:36796] | VIM | Down |
|  | ENSBTAG00000021336 | kinesin family member 5A [Source:VGNC Symbol;Acc:VGNC:30605] | KIF5A | Down |
|  | ENSBTAG00000025219 |  |  | Down |
|  | ENSBTAG00000030198 | transmembrane protein 232 [Source:VGNC Symbol;Acc:VGNC:36046] | TMEM232 | Down |
|  | ENSBTAG00000031814 | serine dehydratase [Source:VGNC Symbol;Acc:VGNC:34399] | SDS | Down |
|  | ENSBTAG00000039154 |  |  | Down |
|  | ENSBTAG00000040290 | glutathione S-transferase omega 2 [Source:VGNC Symbol;Acc:VGNC:29687] | GSTO2 | Down |
|  | ENSBTAG00000045581 |  |  | Down |
|  | ENSBTAG00000046666 | tetratricopeptide repeat domain 9B [Source:VGNC Symbol;Acc:VGNC:36482] | TTC9B | Down |
|  | ENSBTAG00000052924 | sodium/potassium transporting ATPase interacting 2 [Source:HGNC Symbol;Acc:HGNC:16443] | NKAIN2 | Down |
| NFkB | ENSBTAG00000001186 | SH2 domain containing 4A | SH2D4A | Up |
|  | ENSBTAG00000000917 | bone morphogenetic protein 1 | BMP1 | Up |
|  | ENSBTAG00000000625 | Bos taurus SMAD family member 6 (SMAD6) | SMAD6 | Up |
|  | ENSBTAG00000000460 | Bos taurus synaptotagmin like 2 (SYTL2 | SYTL2 | Up |
|  | ENSBTAG00000000376 | purinergic receptor P2X 3 | P2RX3 | Up |
|  | ENSBTAG00000001146 | human immunodeficiency virus type I enhancer binding protein 2 | HIVEP2 | Up |
|  | ENSBTAG00000000016 | Bos taurus lymphotoxin alpha (LTA) | LTA | Up |
|  | ENSBTAG00000000828 | calpain 6 | CAPN6 | Up |
|  | ENSBTAG00000001004 | Bos taurus endothelial cell adhesion molecule | ESAM | Up |
|  | ENSBTAG00000000381 | sphingosine-1-phosphate receptor 4 | S1PR4 | Up |
|  | ENSBTAG00000001468 | sterile alpha motif domain containing 4A | SAMD4A | Up |
|  | ENSBTAG00000001301 | leucine rich repeat containing 32 | LRRC32 | Up |
|  | ENSBTAG00000001864 | nuclear receptor subfamily 4 group A member 3 | NR4A3 | Up |
|  | ENSBTAG00000001401 | Bos taurus solute carrier family 45 member 1 (SLC45A1) | SLC45A1 | Down |
|  | ENSBTAG00000001335 | Bos taurus growth hormone receptor (GHR) | GHR | Down |
|  | ENSBTAG00000000667 | apolipoprotein L, 3 | APOL3 | Down |
|  | ENSBTAG00000000541 | Bos taurus NK2 homeobox 1 (NKX2-1) | NKX2-1 | Down |
|  | ENSBTAG00000000240 | Bos taurus A-kinase anchoring protein 7 (AKAP7) | AKAP7 | Down |
|  | ENSBTAG00000002135 | Bos taurus CD69 molecule (CD69) | CD69 | Down |
|  | ENSBTAG00000008028 | Bos taurus chimerin 1 (CHN1), transcript variant 2, mRNA | CHN1 | Down |
|  | ENSBTAG00000011003 | Bos taurus IKAROS family zinc finger 3 (IKZF3 | IKZF3 | Down |
|  | ENSBTAG00000009126 | Y-box binding protein 2 | YBX2 | Down |
|  | ENSBTAG00000012007 | Bos taurus suppressor of cytokine signaling 2 (SOCS2) | SOCS2 | Down |
|  | ENSBTAG00000008996 | SFI1 centrin binding protein | SFI1 | Down |
|  | ENSBTAG00000008062 | Bos taurus doublesex and mab-3 related transcription factor 2 (DMRT2) | DMRT2 | Down |
|  | ENSBTAG00000008084 | Bos taurus zinc finger protein 382 (ZNF382) | ZNF382 | Down |
|  | ENSBTAG00000009576 | Bos taurus protein kinase C and casein kinase substrate in neurons 3 (PACSIN3) | PACSIN3 | Down |

**Supplementary table 3:** *Theileria annulata* genes with SPs and/or TMD with their orthologs genes in *Theileria parva* and *Theileria orientalis*

| *Theileria annulata* Gene ID | | Product Description | TM Domains | SignalP Peptide | Ortholog count | Computed GO Functions | NLS prediction | Ortholog in T. parva | Product Description | Computed GO Functions | Ortholog in Orientalis | Product Description | Computed GO Functions |
| --- | --- | --- | --- | --- | --- | --- | --- | --- | --- | --- | --- | --- | --- |
| TA02785 | | hypothetical protein, conserved | 0 | MKIILILLIINFVIN | 13 | N/A | N/A | TpMuguga_01g02150 | unspecified product | NA | MACJ_000566 | unspecified product | NA |
| TA03875 | | RNA poly(A)-binding protein, putative | 1 | N/A | 12 | nucleic acid binding | PKRKNIPGYNRRRTNNRT | TpMuguga_03g00088 | Polyadenylate-binding protein 2 | nucleic acid binding | TOT_030000775 | predicted protein | nucleic acid binding, RNA binding |
| TA04835 | | hypothetical protein, conserved | 1 | N/A | 13 | N/A | N/A | TpMuguga_03g00487 | putative integral membrane protein | NA | TOT_030000364 | conserved hypothetical protein | NA |
| TA05280 | | hypothetical protein | 4 | N/A | 8 | N/A | N/A | TpMuguga_03g00125 | putative integral membrane protein |  | TOT_030000760 | conserved hypothetical protein | integral component of membrane |
| TA06810 | | hypothetical protein, conserved | 0 | MYSNRNITVVLLLYITHFVHS | 14 | N/A | N/A | TpMuguga_01g00744 | unspecified product | NA | TOT_010000701 | conserved hypothetical protein | NA |
| TA07230 | | hypothetical protein | 1 | MKIKILFIILIINFIKC | 7 | N/A | N/A | TpMuguga_04g00068 | putative integral membrane protein | NA | TOT_040000825 | conserved hypothetical protein | integral component of membrane |
| TA08280 | | hypothetical protein | 2 | N/A | 8 | N/A | N/A | TpMuguga_04g00092 | putative integral membrane protein | NA | TOT_040000803 | conserved hypothetical protein | integral component of membrane |
| TA08715 | bacterial histone-like protein, putative | 0 | MFTYTNSFLLLIIIICLTVES | 14 | DNA binding | N/A | TpMuguga_04g00110 | Bacterial DNA-binding family protein | DNA binding | TOT_040000787 | uncharacterized protein | DNA binding |  |
| TA09825 | | hypothetical protein, conserved | 1 | N/A | 13 | N/A | N/A | TpMuguga_04g00817 | putative integral membrane protein | NA | TOT_040000111 | conserved hypothetical protein | integral component of membrane |
| TA11950 | | hypothetical P-, Q-rich protein family protein, putative | 1 | MINNIKYLIFVLIFRSCIFVASS | 332 | N/A | N/A | TpMuguga_02g00216 | unspecified product | NA | TOT_030000702 | uncharacterized protein | NA |
| TA20225 | | hypothetical protein | 1 | N/A | 3 | N/A | N/A | TpMuguga_01g00574 | putative integral membrane protein | NA | MACK_000497 | unspecified product | NA |

**Supplementary table 4:** Orthologs genes of *Theileria annulata* annotated in *Theileria parva* and their functions

| Gene ID in *Theileria annulata* | Ortholog gene in *Theileria parva* | Ortholog gene description | Function (GO term) |
| --- | --- | --- | --- |
| TA04760 | TpMuguga_03g00476 | MED6 mediator sub complex component family protein | - regulation of transcription by RNA polymerase II - transcription coregulator activity |
| TA04840 | TpMuguga_03g00488 | 60S ribosomal L34 protein, putative | - translation - ribosome - structural constituent of ribosome |
| TA09695 | TpMuguga_02g00064 | Ribosomal protein S8 family protein | - translation - ribosome - structural constituent of ribosome |

**Supplementary Figure 1:** Host (A) and parasite (B) mapped read count distribution. Black lines show the means; white lines represent individual data points; polygons represent density of the data. **total_reads:** Total clean reads used for analysis, **total_map**: Number and percentage of reads aligned to the genome, **total mapping rate**: (mapped reads)/(total reads)*100, **unique_map**: Number and percentage of reads aligned to the unique position of the reference genome (for subsequent quantitative data analysis), **unique mapping rate**: (uniquely mapped reads)/(total reads)*100, **Multi_map**: number and percentage of reads aligned to multiple locations in the reference genome, **multiple mapping rate**: (multiple mapped reads)/(total reads)*100, **read1_map**: Number and percentage of read1 aligned to the reference genome, **read2_map**: Number and percentage of read2 aligned to the reference genome, **positive_map**: Number and percentage of reads aligned to the positive chain of the reference genome, **negative_map**: Number and percentage of reads aligned to the negative chain of the reference genome, **splice_map**: Number of spliced reads on the genome and its percentage, **unsplice_map**: Number of complete reads aligned to genome and its percentage, **proper_map**: Number of paired read1 and read2 aligned to the genome and its percentage.


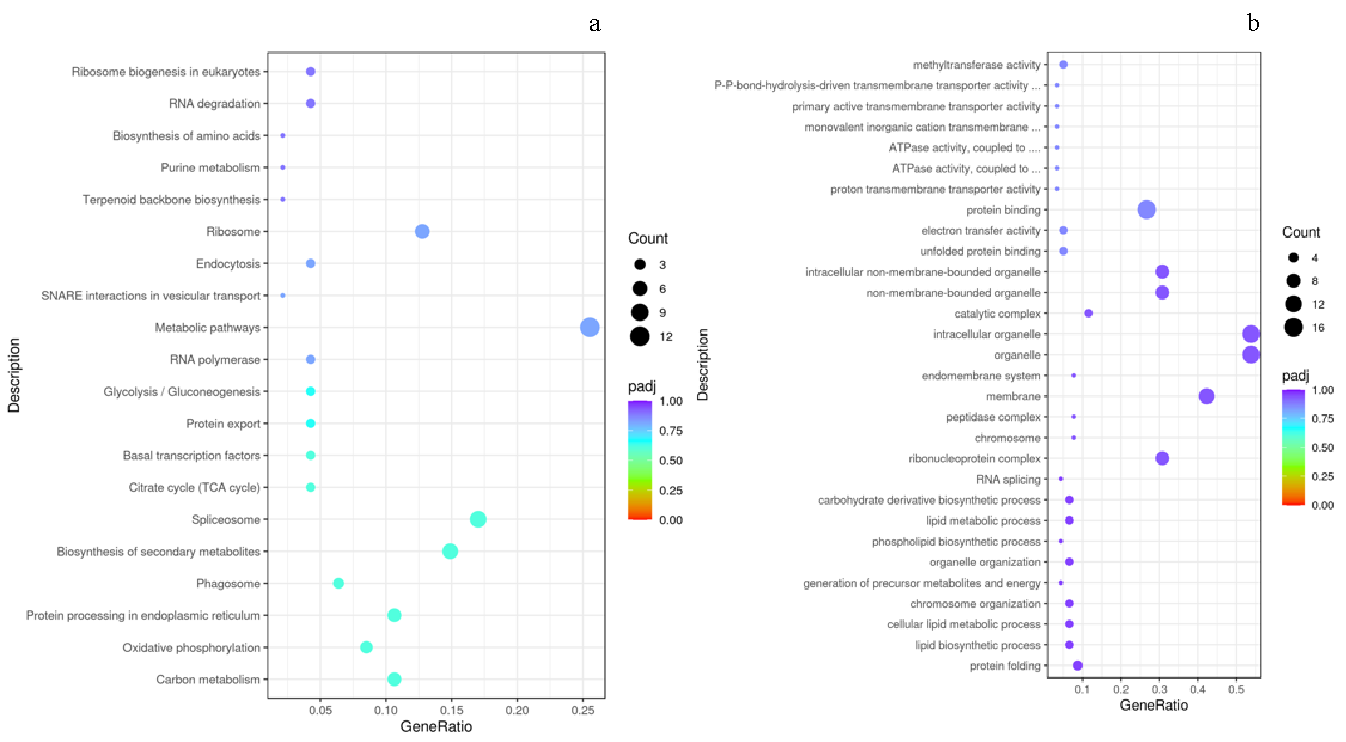


**Supplementary Figure 2.** KEGG (a) and GO (b) enrichment GO Enrichment Analysis Scatter Plot. The abscissa in the graph (a) is the ratio of the number of differential genes on the KEGG pathway to the total number of differential genes, and the ordinate is the KEGG pathway. The abscissa in the graph (b) is the ratio of the differential gene number to the total number of differential genes on the GO Term, and the ordinate is GO Term

**Supplementary Figure 3**. Unique and shared GO enrichment pathways for the most differentially expressed genes in *Theileria annulata* transcript TA04840, TA04760 and TA09695 described as hypothetical proteins
